# Effect of various topical anti glaucoma drugs on cardiopulmonary system. A prospective study

**Published:** 2019

**Authors:** Anu Litoriya, Uma Saran Tiwari, Rachna Pathak

**Affiliations:** * Department of Ophthalmology, Gajra Raja Medical College, Gwalior, India

**Keywords:** spirometry, Timolol, cardio pulmonary side effects of Timolol

## Abstract

**Purpose:** To quantitatively evaluate the cardiopulmonary effects of various topical antiglaucoma drugs.

**Material & method:** In this study, forty consecutive cases of newly diagnosed primary open angle glaucoma were recruited. After taking a detailed history, an ophthalmological examination and a systemic examination including resting pulse rate, blood pressure, ECG, auscultation of the chest and spirometry were performed. Then the patients were randomly divided into four groups and one of the four topical anti glaucoma medication (Timolol, Latanoprost, Brimonidine, and Dorzolamide) prescribed. Patients were reviewed 4 weeks later and the same ocular and systemic examinations were performed.

**Result:** Timolol therapy reduced all the spirometry parameters that are statistically significant difference with the P value of less than 0.1. Timolol therapy resulted in the mean reduction of pulse rate by 3.2 beats/ minute and a mean reduction of systolic and diastolic blood pressure by 5.8 mmHg and 5.6 mmHg, respectively all the spirometry & cardiovascular parameters remained unchanged in the other three groups after 4 weeks of treatment.

**Conclusion:** Timolol significantly affects the cardiopulmonary status. Therefore, we could advice the assessment of cardiopulmonary status mandatory in patients receiving topical beta-blockers. Bronchospasm may be of clinical significance in the elderly, who commonly have undiagnosed reversible airway obstruction.

## Introduction

Glaucoma has its highest prevalence among the elderly population with an incidence of approximately 1% in subjects aged more than 60 years and 3-5% in those between 70 and 80 years [**1**]. Currently, a number of drugs are available in our armamentarium for medical management of glaucoma including beta-blockers, prostaglandin analogue, alpha 2 agonist & carbonic anhydrase inhibitors. Still, the most frequently used drug is the topical non-selective beta-blocker, such as Timolol. A sufficient amount of beta-blockers can be absorbed through the nasopharyngeal mucosa into the systemic circulation, thereby potentially causing bradycardia & respiratory impairment [**2**]. These changes, particularly bronchospasm may be of clinical significance in the elderly, who commonly have undiagnosed reversible airway obstruction [**3**].

Hence, this prospective interventional study was conducted to quantitatively evaluate the cardiopulmonary effects of various topical antiglaucoma drugs.

## Method

This prospective interventional study was undertaken in the Department of Ophthalmology of our tertiary care hospital after obtaining clearance from the institutional ethical committee. Forty consecutive cases of newly diagnosed primary open angle glaucoma (POAG) attending the glaucoma clinic were recruited in this study. A subject having PACG, secondary glaucoma, pre existing pulmonary problems such as Bronchial asthma, Chronic obstructive pulmonary diseases, cardiac problems such as bradycardia with resting pulse rate less than 60 beats per minute, those with clinical evidence of heart failure, arrhythmia & heart blocks, were excluded. A written informed consent was obtained from the participants. Demographic details and a detailed history of the participant were recorded. Detailed ophthalmologic examination was carried out. Systemic examination including resting pulse rate, blood pressure (BP), electrocardiogram (ECG), auscultation of the chest and spirometry, were performed. Spirometry parameters included Peak expiratory flow rate (PEFR), Forced expiratory volume in 1 second (FEV1), and forced vital capacity (FVC), FEV1/ FVC%). Patients were randomly divided into four groups according to the topical antiglaucoma medications used as monotherapy i.e. Timolol, Latanoprost, Brimonidine and Dorzolamide groups. Patients were followed after 4 weeks to evaluate the cardio pulmonary status using the above-mentioned parameters with special reference pulse, BP, and spirometry. 

## Results

Forty participants in this study included 25 males and 15 females aged between 60 and 80 years. The mean age of participants was 62.5+/ -15.7 years. The data on spirometry were compiled and analyzed (Table 1, 2). The mean values of all the four spirometry parameters (FEV1, FVC, PEFR) and cardiovascular parameters fell after 4 weeks of therapy in Timolol group, showing a statistically significant difference with the P value of less than 0.1 (**Table 1**,**2**).

**Table 1:** Mean values of spirometry parameters before and after 4 weeks of therapy

Drugs	FEV1			FVC			PEFR			FEV1/ FVC %	
	Before	After	p - value	Before	After	p - value	Before	After	p - value	Before	After
Timolol	2.43 ± 0.506497	2.07 ± 0.465685	0.11	3.07 ± 0.695734	2.77 ± 0.586595	0.31	4.97 ± 0.565313	4.23 ± 0.353403	0.002	82.24%	72.41%
Latanoprost	1.97 ± 0.339085	1.98 ± 0.33286	0.94	2.74 ± 0.477256	2.72 ± 0.493794	0.92	4.54 ± 0.245196	4.52 ± 0.265926	0.86	72.96%	74.12%
Brimonidine	2.63 ± 0.43765	2.69 ± 0.437047	0.76	2.85 ± 0.45976	2.82 ± 0.460319	0.88	5.04 ± 0.591781	5.04 ± 0.580388	1.00	92.20%	91.70%
Dorzolamide	2.67 ± 0.414087	2.62 ± 0.41313	0.78	2.75 ± 0.536756	2.70 ± 0.399972	0.81	4.82 ± 0.519333	4.79 ± 0.629907	0.90	85%	84.02%
*FEV1 = Forced expiratory volume at 1 minute, FVC = Forced vital capacity, PEFR = Peak expiratory flow rate*											

**Table 2:** Mean values of cardiovascular parameters before and after 4 weeks of therapy

Drugs	Heart rate (beats/ minute)				Systolic blood pressure (mmHg)				Diastolic blood pressure (mmHg)			
	Before	After	Difference	p - value	Before	After	Difference	p - value	Before	After	Difference	p - value
Timolol	73.8 ± 1.988858	70.6 ± 1.349897	-3.2	0.0005	132.8 ± 9.189366	127.0 ± 8.011103	-5.8	0.14	85.0 ± 6.74948	79.4 ± 4.714045	-5.6	0.04
Latanoprost	74 ± 2.394438	73.9 ± 2.983287	-0.1	0.93	132.0 ± 4.848826	130.8 ± 6.719788	-1.2	0.45	84.0 ± 5.27046	83.0 ± 4.830459	-1.0	0.66
Brimonidine	74.3 ± 2.953341	73.9 ± 3.979112	0.4	0.80	137.0 ± 5.163978	136.0 ± 5.202563	-1.0	0.67	85.0 ± 5.2704	83.4 ± 3.794733	-1.8	0.44
Dorzolamide	74.0 ± 2.406011	74.0 ± 1.95789	0	1.00	120.6 ± 6.25744	120.0 ± 6.425643	-0.6	0.83	80.0 ± 6.74619	80.0 ± 5.370702	0	1.00

## Discussion 

In our study, Timolol was found to be associated with a mean reduction of the resting pulse rate and a mean reduction of the systolic and diastolic blood pressure respectively. This is in accordance to previous studies, whose authors found a mean reduction in cardiopulmonary parameters in Timolol group in their study [**6**,**7**]. The same findings have been reported by Watson et al., who found a mean reduction of 2 beats/ minute in the Timolol group and a decrease in blood pressure. None of the drugs other than Timolol in this study was found to affect the cardiovascular parameters significantly [**12**].

Timolol reduced all spirometry parameters significantly. All the spirometry parameters were almost unchanged in the rest of the three groups, and on repeated examination at the end of four weeks similar results were found by other authors [**7**-**11**]. 

This study confirmed that significant amounts of topically applied eye drops reach the systemic circulation and so, cardiovascular and/or respiratory side effects may result in elderly POAG patients, even if they are screened for cardiac and respiratory diseases. Because 80% of the volume of the eye drop (approximately 60 micro liters) is drained into the lacrimal pathway, about 1 to 1.2 mg of timolol can be absorbed daily by the highly vascularized mucosa of the nasolacrimal ducts [**3**]. This amount is far lower than the oral dose of beta-blockers i.e. 20-60 mg/ day for the treatment of hypertension [**4**,**13**]. However, oral beta-blockers undergo substantial “first pass hepatic metabolism”, with the actual dose that reaches the circulation being less than 10% of the amount absorbed by the gastrointestinal tract [**3**]. Because the eye drop is immediately absorbed by the mucosal vasculature, the liver is bypassed. Therefore, by avoiding first pass hepatic metabolism, higher drug plasma levels relative to their starting dose is achieved, which may explain the systemic side effects of topical beta blockers despite low doses [**14**]. 

Reversible airway obstruction is a cautionary indication to the use of beta-blocker and the after mentioned value has been deemed to necessitate a change in medication in previous studies (Diggory P & Cassel Brown et al.) [**5**,**11**]. This study supports the conclusion of previous workers that many people suffer from unrecognized respiratory impairment when prescribed topical beta blockers & by starting beta blocker therapy and repeating it after 1 month, most patients developing respiratory impairment will be identified. Therefore, it would seem prudent to evaluate any new glaucoma patient who will be using a topical beta-blocker with spirometry.

## Conclusion

Topical beta-blockers do reduce the respiratory “reserve” of patients. Timolol lowers resting pulse rate (3.2 beats/ minute) and blood pressure (5.6 mmHg). Finally, this study reiterates previous work, advising the assessment of cardiopulmonary status mandatory in patients receiving topical beta-blockers for the control of their glaucoma. 
